# A Physical Analog to Assess Surgical Face Mask Air Flow Resistance During Tidal Ventilation

**DOI:** 10.3389/fphys.2022.808588

**Published:** 2022-02-17

**Authors:** Bruno Demoulin, Claude Duvivier, François Marchal, Silvia Demoulin-Alexikova

**Affiliations:** ^1^EA 3450 DevAH, Université de Lorraine, Vandœuvre-lès-Nancy, France; ^2^Service des Explorations Fonctionnelles Respiratoires, CHU Lille, Lille, France; ^3^CNRS, Inserm, CHU Lille, Institut Pasteur de Lille, U1019-UMR9017-CIIL-Centre d′Infection et d′Immunité de Lille, University of Lille, Lille, France

**Keywords:** face masks, surgical masks, breathability, COVID-19, SARS-CoV-2, pressure-flow relationship, tidal breathing, airway resistance

## Abstract

A large variety of disposable face masks have been produced since the onset of the COVID-19 pandemic. Decreased resistance to inspiration improves adherence to the use of the mask; the so called breathability is usually estimated by the measurement of air flow across a section of the tissue under a given pressure difference. We hypothesized that the mask pressure—flow relationship studied in conditions that mimic tidal breathing could allow a more comprehensive characterization of airflow resistance, a major determinant of mask comfort. A physical analog was made of a plaster cast dummy head connected through a pneumotachograph to a series of bellows inflated/deflated by a respirator. Pressure was measured at the mock airway opening over which the mask was carefully secured. The precision of the measurement equipment was quantified using two estimates of measurement error: repeatability coefficient (RC) and within-mask coefficient of variation (CV_wm_). The airflow resistance of 10 surgical masks was tested on 4 different days. Resistance means did not differ significantly among four repeated measures (0.34 hPa.s.L^−1^; 0.37 hPa.s.L^−1^; 0.37 hPa.s.L^−1^; and 0.37 hPa.s.L^−1^; *p* = 0.08), the estimated RC was 0.08 hPa.s.L^−1^ [95%CI: 0.06–0.10 hPa.s.L^−1^], and CV_wm_ was 8.7% [95%CI: 1.5–12.2%]. Multiple comparisons suggest the presence of a learning effect by which the operator reduced the error over the course of repetitive resistance measurements. Measurement precision improved considerably when the first set of measures was not taken into account [RC ~ 0.05 hPa.s.L^−1^ (95%CI: 0.03–0.06 hPa.s.L^−1^); CV_wm_~4.5% (95%CI: 1.9–6.1%)]. The testing of the face mask resistance (R) appears simple and highly repeatable in conditions that resemble tidal breathing, once operator training was assured. The procedure adds further to the current standard assessment of breathability and allows estimating the maximal added respiratory load, about 10–20% of the respiratory resistance reported in heathy adult subjects.

## Introduction

The SARS-CoV-2 pandemic has led to the generalized use of face covering materials to minimize respiratory transmission of the disease. Saliva droplet projection was identified as the major route for respiratory transmission for which surgical masks appear to provide equally efficient protection compared to face piece respirators, although with furthering the knowledge in COVID mechanisms, aerosols may also represent a possible route especially at the bedside of COVID patients, where ambient air may contain high concentration of viral particles (Sommerstein et al., 2020). In mask design, one attempts to determine an optimal compromise between efficient particulate filtration and ease of wear, referred to as breathability (Aydin et al., 2020; Ju et al., 2021), which is related to the added respiratory load. Breathability of surgical face masks usually is estimated *in vitro* from the pressure drop across a given section of the filtering tissue under conditions of unidirectional, constant air flux (Forouzandeh et al., 2021), on which recommendations are based. The end point in conceiving any protection material is optimal compromise between filtering efficiency and breathability.

During tidal breathing, the added resistance may vary with flow amplitude or direction; however, we are aware of little characterization of such properties. It would also be helpful to be able to quantify the magnitude of the added maximal load as a fraction of the subject’s respiratory resistance. The fact that, in the long term, mask comfort significantly contributes to a subject’s adherence to its use justifies more detailed studies of mask mechanics under conditions that resemble tidal breathing.

Such evaluation requires a set up under which the mask can be subjected to rhythmic flow changes that mimic tidal breathing, while pressure and flow are being measured. This allows the determination of mask resistance to breathing. Once validated under controlled conditions with the reference surgical face mask, we surmised that this set up could be used to test any type of face protection. This is of special interest in view of the initial non-reusable masks shortage during the first months of the SARS-CoV-2 pandemic (Lepelletier et al., 2020; Maher et al., 2020; Forouzandeh et al., 2021) that prompted the production of a variety of face protection. A recent paper proposed a computerized system to study mask breathability on a dummy head in dynamic conditions. The dynamic pressure difference across the mask was used to compare different types of materials (Yao et al., 2019). The current study is intended to go a step further, i.e., to express the mask resistance by relating pressure to flow so as to have the potential to describe its time course, flow dependence, and magnitude relative to the subject’s own respiratory resistance.

The aim of this study was to determine the characteristics of surgical mask resistance using a physical analog under conditions that resemble physiological breathing and to quantify its precision under the same operating conditions over a short interval of time (repeatability). More specifically, it was intended to validate a model set up closer to real life than the current reference procedure measuring a pressure drop generated across a fabric surface at constant, unidirectional flow. This approach would also allow to estimate the maximal load to breathing offered by the mask as a fraction of the total respiratory resistance. During the tests, the mask covered the airway opening of a dummy head; standard measuring conditions, minimal leakage, and optimal reproducibility were insured by tightly fitting the mask to the cast. The resistance of a mask tissue section was also tested in order to assess breathability in conditions similar to the standard procedure (Forouzandeh et al., 2021).

## Materials and Methods

### Physical Analog

**Figure 1:**
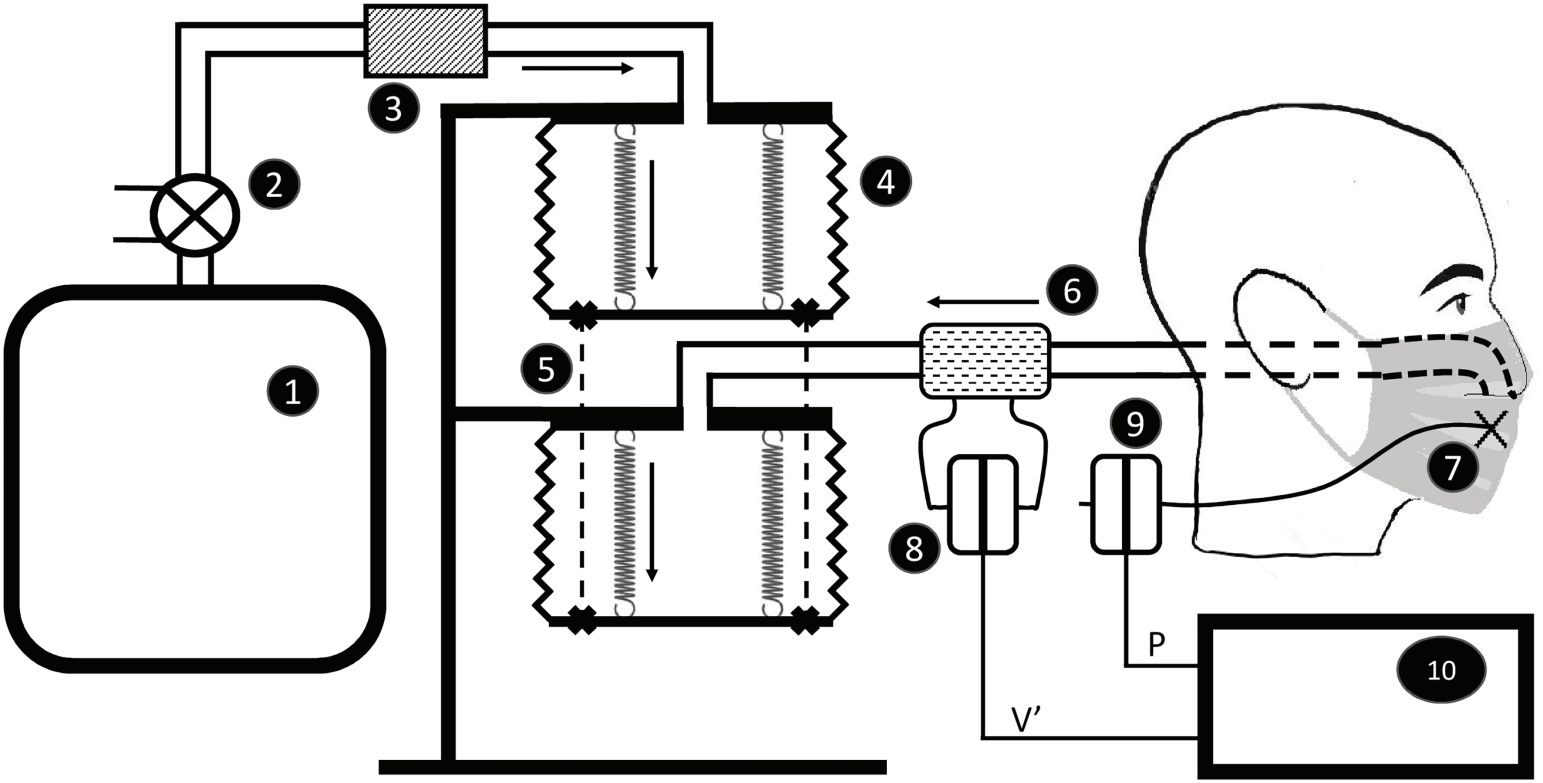
Sketch of the apparatus to measure resistance of the face mask secured on the dummy head. The respirator (1) is connected through a valve (2) and a resistor (3) to bellows (4) attached through rails and springs (5). The second bellow is connected through a pneumotachograph (6) to the mock airways. Pressure is measured at four different points behind the mask (7). Flow (8) and pressure (9) signals are fed to a chart lab recorder (10). Dark arrows indicate the direction of flow during inspiration.

The device is illustrated in Figure 1. The mask support system consists of a plaster cast dummy head. Nostrils and mouth were connected from behind to a time cycled, pressure limited respirator (O’nyx Plus Pierre Medical SA, France) that delivers airflow to a set of identical bellows mechanically coupled through rails and springs. Both bellows exhibit the same excursion, hence identical volume change. Valve devices triggered by the respirator determine bi-directional flow, mimicking inspiration and expiration. The respirator is set to deliver a peak pressure of 40 hPa at a frequency of 25 cycle.min^−1^ and a peak flow of 1.5 L.s^−1^ (3 L.s^−1^ peak to peak). Experiments were performed under ambient conditions of pressure (950 hPa), temperature (22°C), and relative humidity (40%).

Pressure was measured at four different points behind the mask using a Honeywell 176 PC 14HD1 transducer previously calibrated using a slanted manometer. Flow was measured in the circuit close to the plaster head (Figure 1) using a Fleisch # 2 pneumotachograph (Metabo Lausanne Suisse; Fleisch, 1956). The device is linear within 5 L.s^−1^ peak to peak. The flowmeter was attached to an identical pressure transducer and calibrated by the integral method (Varene et al., 1974). The frequency response of both transducers is matched within 1% of amplitude and 2° of phase up to 30 Hz (Duvivier et al., 1991). Pressure (P) and flow (V’) signals were sampled at a frequency of 40 Hz and passed through a digital band pass filters (0.1–5 Hz) and fed to a lab-chart recorder (Power Lab 16/30 AD Instruments United States). The signals were continuously displayed on a screen during the acquisition period and stored on disk for later analysis.

### Protocol

A set of 10 surgical face masks (Foshan Xinbao Technology Co. Ltd., China, Zhejiang Longde Pharmaeutical Co. Ltd., China) was tested on the dummy head on 4 separate days by the same operator. The order of measurements was randomly determined prior to the study and kept throughout. The mask was applied to cover nose and mouth, the flexible metallic edge pinched over the nose bridge, and elastic bands adjusted around each ear lobe and retracted together at the back. The mask contours were then carefully secured on the plaster using adhesive tape (3M Micropore Professional Care 3M Deutschland GmbH). The mask tissue surface area available to airflow was 180 cm^2^. Trials were first performed to examine mask contours for gross air leaks. Thereafter, the P – V′ acquisition was tracked for at least 1 min.

### Data Analysis

#### Resistance Computation

**Figure 2:**
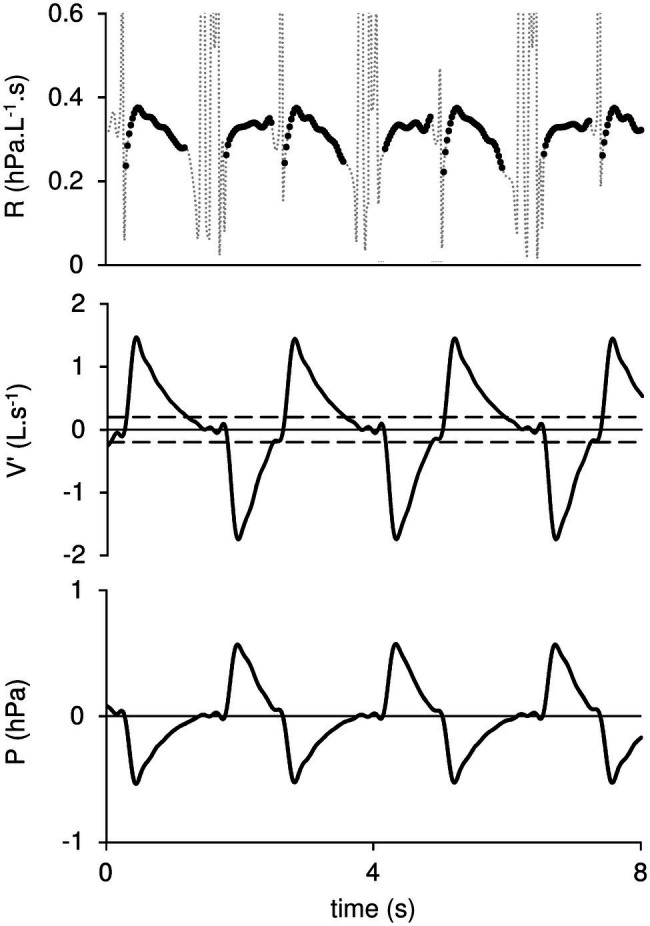
Mask resistance (R), Flow (V′), and pressure (P) are plotted against time. The dotted lines on R tracing correspond to data rejected from the computation. Horizontal broken lines on V′ indicate the −0.2 L.s^−1^ to +0.2 L.s^−1^ interval beyond which R values are rejected. Positive V′ values indicate inspiration.

Each acquisition period included more than 2,400 sets of P and V′. The mask resistance (R) was computed from each set as the ratio of P to V′. Those V′ values ranging from −0.2 to +0.2 L.s^−1^ were filtered out, since R computations using V′ close to 0 generated artefacts (e.g., Figure 2).

Among different models used to describe airway/respiratory resistance, a convenient and valuable expression relates R to V′ by linear regression (Peslin et al., 1992). This empirical approach allows to take into account both linear (K1) and non-linear (K2) components of R such that:


R=K1+K2×|V′|

K1 may also be known as the resistance extrapolated at zero flow and K2 includes its flow dependent component, if any. The latter may frequently be neglected under low ventilation regimen but may be worth taking into account when ventilation is increased such as during exercise or cough. The analysis was performed on the whole data set as well as separately on inspiration and expiration using a Borland C++ program specifically developed to correlate resistance to flow.

#### Statistics

Statistical analysis was performed using Prism (GraphPad Software, LLC). For a given trial, the mean values of R, K1, and K2 were obtained. All intermediate calculations were carried out to full precision and rounded to three decimal places at the reporting stage. In order to estimate precision of mask resistance measurement performed by equipment, several estimates of measurement error have been assessed. Repeatability coefficient (RC) was used to express the precision in dimensional and within-mask coefficient of variation (CV_wm_) in non-dimensional terms.

Repeatability coefficient was calculated from the within-mask variance estimated using repeated measures ANOVA. It is the maximum difference that is likely to occur between repeated measurements which is defined by


$$1.96\times \surd 2\times s_{w}$$

Where s_w_ is the within-mask SD, a square root of within-mask variance (Bland and Altman, 1996; Bartlett and Frost, 2008).

In order to check whether the measurement errors for each mask do not depend on the magnitude of the measurement, we performed Kendall correlation between SD for each mask (SD_m_) and each mask mean (Mean_m_). Assumption of sphericity was checked using Mauchly’s test. In the case of violation of sphericity assumption, the degrees of freedom and *p*-value were adjusted using Greenhouse–Geisser epsilon correction. The normality of residuals was tested using the Kolmogorov–Smirnov test. In the case of violation of normality assumption, RC was calculated from within subject variance estimated from repeated measures of ANOVA, but CIs for RC were calculated using bootstrapping technique (Bartlett and Frost, 2008).

CV_wm_ was calculated using the root mean square method (Hyslop and White, 2009) as


$$CV_{wm}\;\left(\%\right)=100\times \surd \left[\frac{1}{n}\sum CV_{m}^{2}\right]$$

Where CV_m_^2^ is the squared coefficient of variation of each mask’s repeated measurements and *n* is the number of repeated measurements.

The effect of V′ direction was estimated by comparing respectively R, K1, and K2 between inspiration and expiration using Student paired *t*-test.

### Additional Experiment: Tissue Airflow Resistance and Breathability

At the end of repeated measurements of mask resistance, breathability was also assessed under conditions similar to the standard procedure. A section of each mask was tightly fitted to a circular—26 cm^2^—support, so that the measurement could be performed on the fabric under leak proof conditions. The support was connected to the respirator circuit in place of the dummy head and the acquisition performed as previously described. A statistical comparison was performed between this measurement and the 4th series of whole masks, after correcting for the estimated surface area available to flow (180 cm^2^).

## Results

**Figure 3:**
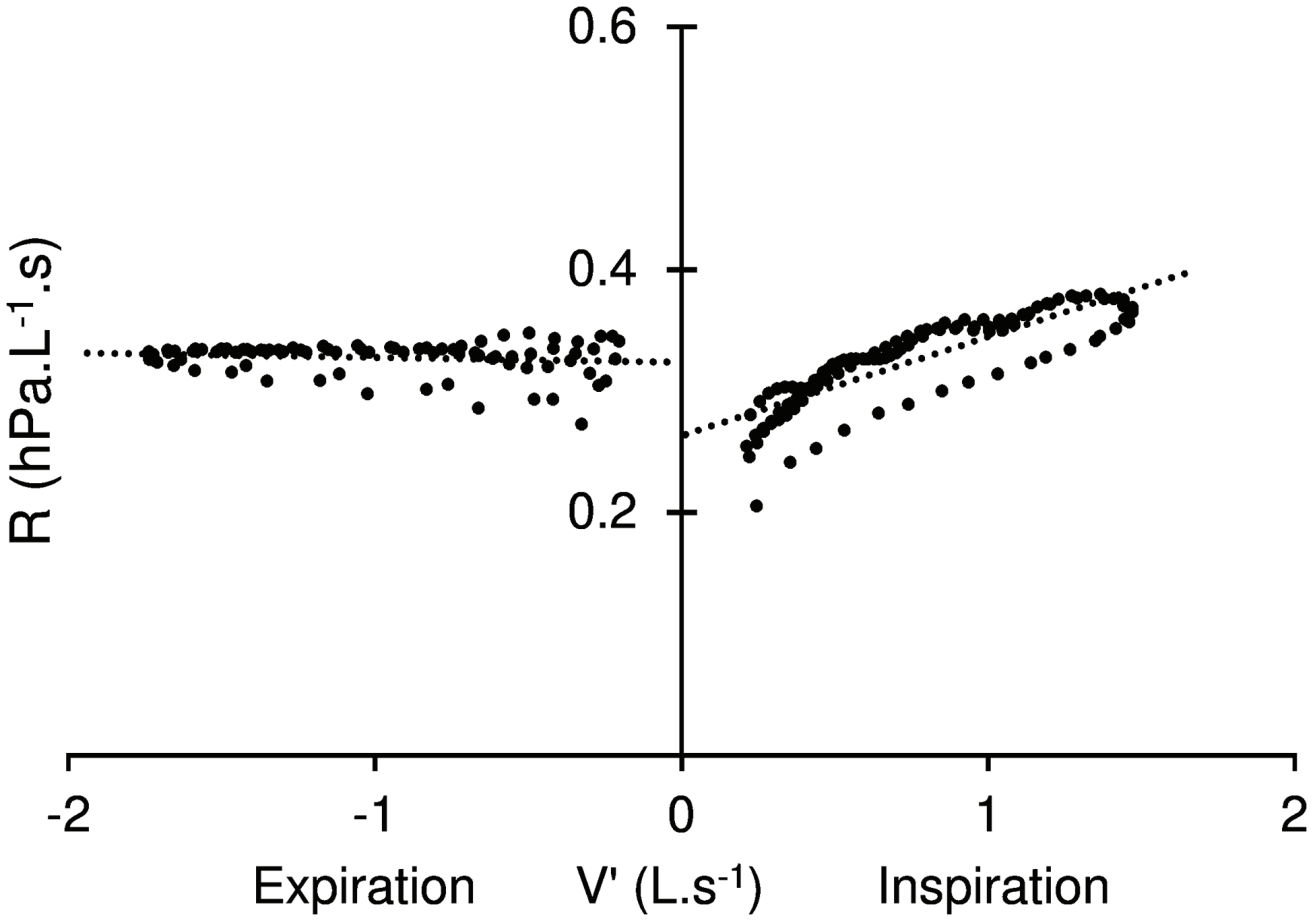
Resistance (R) – Flow (V′) diagram. Positive flow dependence, more apparent in inspiration (positives values of V′) than expiration is indicated by dotted line. K1 (the resistance extrapolated at zero flow) is represented by the intersection with V′ axis. Also note some looping in the relationship during both inspiration and expiration.

P, V′, and R are plotted against time in Figure 2. The dotted lines in the R tracing indicate values discarded from the computation, i.e., corresponding to the V′ interval from −0.2 L.s^−1^ to +0.2 L.s^−1^. R is plotted against V′ in Figure 3. Positive flow dependence is also indicated, mainly in inspiration. In addition, some looping between R and V′ is apparent in both inspiration and expiration.

The mask resistance data are summarized in Table 1. There were no evidence of relationship between measurement error and the magnitude of the measurement of each mask (Kendall’s *τ* = −0.25; *p* = 0.082). Overall residuals were normally distributed and Greenhouse–Geisser epsilon correction was applied as sphericity assumption was violated.

**Table 1:** Repeated measurements of mean resistance (hPa.s.L^−1^) for 10 masks (Set 1–4) and their tissue airflow resistance (Tissue).

Mask #	Tissue	Set 1	Set 2	Set 3	Set 4	Mean_m_	SD_m_	CV_m_
1	0.475	0.431	0.427	0.420	0.402	0.396	0.013	0.033
2	0.426	0.281	0.390	0.430	0.387	0.387	0.064	0.164
3	0.422	0.408	0.409	0.390	0.404	0.384	0.008	0.022
4	0.432	0.304	0.413	0.388	0.357	0.379	0.047	0.124
5	0.372	0.395	0.400	0.393	0.386	0.386	0.006	0.0153
6	0.382	0.351	0.387	0.342	0.402	0.387	0.0314	0.081
7	0.376	0.404	0.395	0.387	0.394	0.338	0.007	0.021
8	0.368	0.214	0.296	0.298	0.313	0.295	0.045	0.152
9	0.351	0.297	0.325	0.312	0.302	0.311	0.013	0.041
10	0.396	0.308	0.311	0.312	0.324	0.338	0.007	0.021
Mean_s_	0.400	0.338	0.375	0.367	0.367	0.360	0.024	0.067
SD_s_	0.038	0.070	0.047	0.047	0.040	N/A	N/A	N/A

Each row corresponds to the repeated measures of one mask. Mean_s_ (SD_s_) = mean (SD) of each set; Mean_wm_ (SD_wm_, CV_wm_) = mean (SD, CV) of each mask, calculated from set 1–4, calculated from set 1. All intermediate calculations were carried out to full precision and rounded to three decimal places at the reporting stage.

Repeated measures ANOVA did not show a significant difference among the four sets of measures (*F* = 3.1; *p* = 0.085). The estimated within-mask variance and s_w_ were 0.0008 hPa.s.L^−1^ and 0.028 hPa.s.L^−1^, respectively. The value of RC was 0.078 hPa.s.L^−1^ [95%CI: 0.058–0.098 hPa.s.L^−1^] and that of CV_wm_ was 8.734% [95%CI: 1.535–12.255%].

Multiple comparisons were performed in order to analyze all paired differences between resistance means. As can be seen from Table 2, the absolute mean differences between 1st set and any subsequent set are at least 3.5-fold greater compared to paired differences among sets 2 to 4.

**Table 2:** Pairwise comparisons matrix for resistance measurements.

Paired comparisons	Mean 1	Mean 2	Mean difference	95% CI of mean difference
Set 1 vs. Set 2	0.339	0.375	−0.036	−0.08225 to 0.01055
Set 1 vs. Set 3	0.339	0.367	−0.028	−0.08446 to 0.02852
Set 1 vs. Set 4	0.339	0.367	−0.028	−0.07444 to 0.01925
Set 2 vs. Set 3	0.375	0.367	0.008	−0.01351 to 0.02926
Set 2 vs. Set 4	0.375	0.367	0.008	−0.01398 to 0.03048
Set 3 vs. Set 4	0.367	0.367	0.0004	−0.02804 to 0.02879

When calculating the precision estimates from set 2–4, the estimated within-mask variance and s_w_ were 0.0003 hPa.s.L^−1^ and 0.017 hPa.s.L^−1^. The value of RC was 0.047 hPa.s.L^−1^ [95%CI: 0.032–0.062 hPa.s.L^−1^] and that of CV_wm_ was 4.525% [95%CI: 1.906–6.108%].

The use of a mask tissue section showed significantly higher resistance compared to the 4th set of repeated measures (*t* = 2.7; *p* = 0.024).

When computing mask resistance separately during the two phases of the V′ cycle for sets 2–4, both R and K1 were found slightly but systematically lower in inspiration compared to expiration that resulted in a significant difference (R: *t* = −9.8; *p* = 0.00001, K1: *t* = −14.9; *p* = 0.0001) of these variables between the two phases of the respiratory cycle (Table 3). Concerning K2, this variable was significantly higher in inspiration compared to expiration (*t* = 21.4; *p* = 0.0000001).

**Table 3:** The mask resistance (R), the mask resistance extrapolated at zero flow (K1) and flow dependent component of resistance (K2) during inspiration and expiration, expressed as mean calculated from set 1–4.

Mask #	R		K1		K2	
	Inspiration	Expiration	Inspiration	Expiration	Inspiration	Expiration
1	0.419	0.422	0.345	0.396	0.104	−0.026
2	0.370	0.375	0.289	0.351	0.109	−0.025
3	0.401	0.405	0.322	0.381	0.105	−0.024
4	0.363	0.369	0.287	0.355	0.094	−0.014
5	0.392	0.395	0.329	0.372	0.088	−0.023
6	0.369	0.373	0.303	0.355	0.090	−0.019
7	0.394	0.397	0.326	0.374	0.084	−0.023
8	0.279	0.282	0.221	0.264	0.082	0.018
9	0.306	0.312	0.242	0.312	0.085	−0.0003
10	0.312	0.316	0.251	0.308	0.083	−0.008
Mean	0.360	0.365^*^	0.291	0.347^*^	0.092	−0.018^*^
SD	0.046	0.046	0.042	0.040	0.010	0.008

All intermediate calculations were carried out to full precision and rounded to three decimal places at the reporting stage.

* R, K1, and K2 inspiration vs. expiration *p* < 0.0001.

## Discussion

Surgical mask air flow resistance has been measured on a dummy head under conditions that simulate quiet tidal breathing in an adult subject. Estimated precision of mask resistance measurement appear satisfactory, at least once adequate sealing procedure was assured. In fact, the first series R was lower than any of the further sets that all recovered the same 0.37 hPa.L^−1^.s value.

The model described here resembles the Sheffield dummy head that has been developed for the validation of filtering face pieces and respirators in the context of airway protection of workers in dusty environments (Mogridge et al., 2016). To the best of our knowledge, the standard testing for tissue face mask breathability measures the pressure drop across a given tissue surface area, while a constant air flow is passed through (Konda et al., 2020; Zangmeister et al., 2020; Cortes et al., 2021; Forouzandeh et al., 2021). According to guidelines currently available in this country (AFNOR SPEC S76-001:2020), breathability should correspond to a flow at least 96 L.s^−1^ through a tissue area of 1 m^2^ under a differential pressure of 1 hPa; that is, a resistance of 104 hPa.s.L^−1^.cm^−2^. From the current average measurements (Table 1), the resistance would be 65.2 hPa.s.L^−1^.cm^−2^ for the mask tested *in situ* and 72.0 hPa.s.L^−1^.cm^−2^ for the isolated tissue. Corresponding breathability would be 153.35 L.m^2^.s^−1^ and 138.9 L.m^2^.s^−1^, respectively. Both estimates are largely above the recommended threshold (96 L.m^2^.s^−1^), but the whole mask value is significantly larger. This could be explained by small air leaks still occurring with measurement on the dummy head, despite the great care taken to insure optimal adhesion. Another possible explanation would be related to some imprecision in determining the exact mask surface area available to airflow on the model. Alternatively, a different dynamic behavior between tissue alone and whole mask during the measurement could help explain the finding, as developed below.

The average 0.37 hPa.s.L^−1^ mask resistance measured here during simulated tidal breathing would represent an increased airway resistance of 20.6 and 16.8%, respectively in healthy adult males and females, based upon recent plethysmographic measurements (Koch et al., 2013). A recent plethysmographic study in a similar population of healthy adults found an almost doubling of the airway resistance measured through a surgical mask (Lassing et al., 2020). For the sake of measuring conditions, however, the airway opening was connected to the breathing apparatus through a rigid face mask, likely excluding a significant surgical mask area available to airflow, hence magnifying the total airway resistance. Nevertheless, that the surgical mask resistance may impede breathing is also supported by measurements of FEV1 (Fikenzer et al., 2020). We are unaware of further direct assessment of airway resistance when breathing through a surgical face mask, but *in vivo* measurements using rhinomanometry and rhinospirometry in healthy subjects breathing through N95 respirators demonstrated a doubling of the nasal resistance (Lee and Wang, 2011). The data are in keeping with an airway pressure of 2–5 hPa reported at a peak flow of 1.4 L.s^−1^ in subjects breathing through respirators masks (Louhevaara, 1984).

Performing repeated measurements of mask resistance using the current analog highlights several important points. Assuming no difference in resistance between 10 surgical masks from the same manufacturer, the estimates of repeatability should reflect precision of the measurements, provided they are repeated under the same conditions (Bland and Altman, 1999; Hyslop and White, 2009). The maximum difference that is likely to occur between four repeated mask resistance measurements (RC) was estimated to be 0.08 hPa.s.L^−1^ [95%CI: 0.06–0.1 hPa.s.L^−1^] and the CV_wm_ was estimated to be 9% [95%CI: 1.5–12.2%] pointing to the fact that the precision of the measurement process was satisfactory. However, analysis of all paired comparisons between resistance measures revealed that: (a) absolute differences between 1st set of measures are considerably higher (at least 3.5-fold) compared to any subsequent set and (b) all mean differences between 1st and any subsequent set were negative (Table 2). This suggests the existence of a learning effect by which the operator reduced the error over the course of repetitive resistance measurements. Indeed, measurement precision improved considerably when the first set of measures was not taken into account in the calculation of repeatability [RC ~ 0.047 hPa.s.L^−1^ (95%CI: 0.03–0.06 hPa.s.L^−1^); CV_wm_ ~ 4.5% (95%CI: 1.9–6.1%)]. Altogether, these results suggest that a great part of measurement error was operator-related, i.e., caused by imperfections in the sealing procedure. Owing to the fact that the mean resistance of the first series was lower than any further set (Table 1) and the mean difference between 1st and any subsequent was negative (Table 2), it is suggested that imperfections in the sealing procedure resulted from air leaks occurring during measurement. These observations highlight the requirement for a mask fixation training.

**Figure 4:**
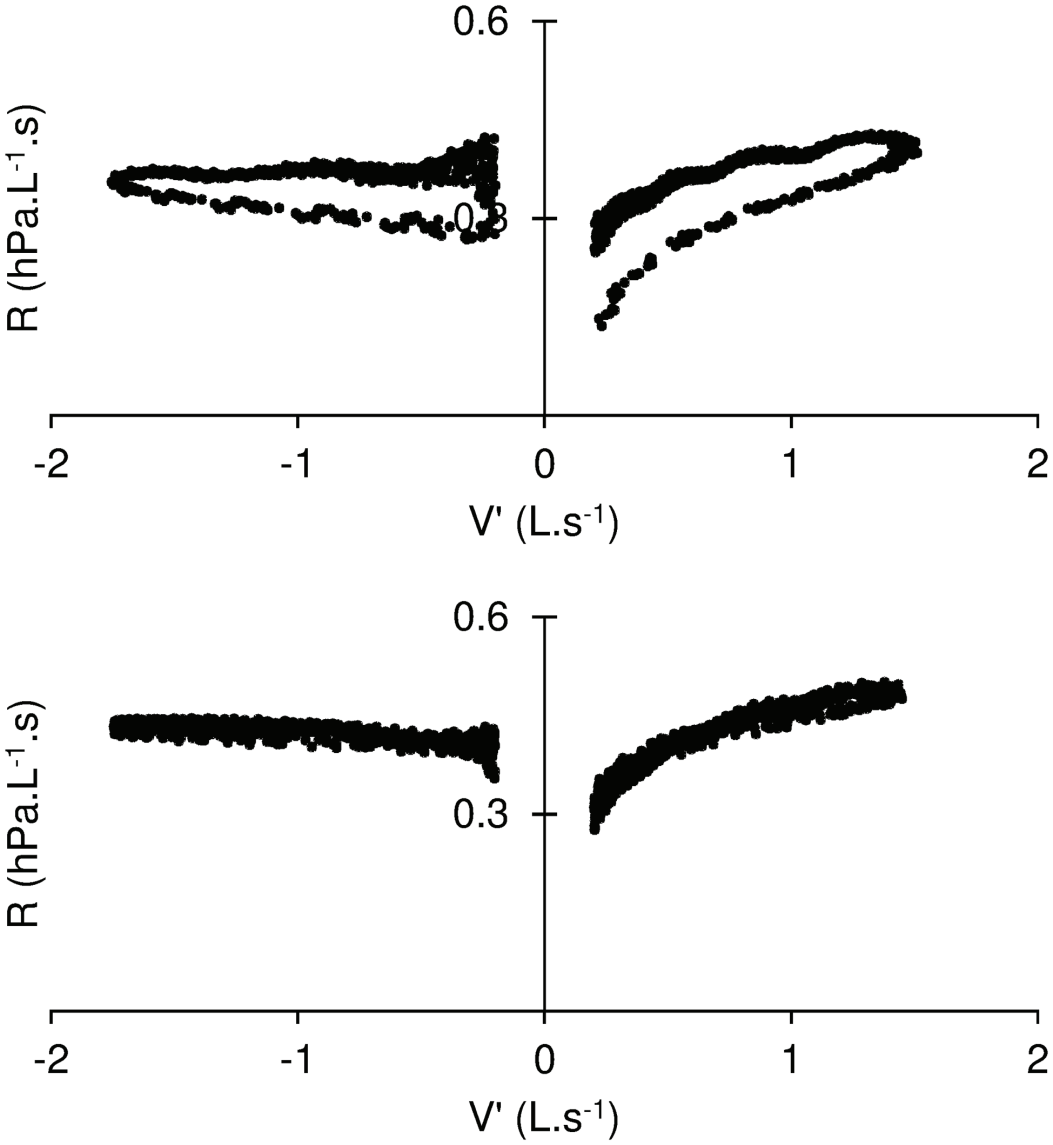
Resistance (R) – Flow (V′) diagram during standard measuring conditions (top) and while the mask motion is prevented by manually holding its edges. The manoeuver is associated with disappearance of the looping of R vs. V′.

With the current analog, a careful examination of the pressure flow relationship can be done under conditions that resemble tidal breathing. The difference between inspiration and expiration was significant for the resistance, even more so for K1, the resistance extrapolated at zero flow (Table 3). In addition, some degree of looping of the resistance flowdiagram—such as shown in Figure 3—was usually apparent. These observations, together with the significant K2 difference between inspiration and expiration may appear somewhat counterintuitive, should flow be the sole determinant to the time variation of resistance. In fact, some change in mask shape and surface area was usually detectable—although to a variable extent—throughout the simulated breath. Most noticeable was the sudden bulging at onset of expiration, with the reverse motion in inspiration being somewhat limited by contact with the plaster cast. In fact, when the mask excursion was minimized by manually holding its edges, both looping and difference between inspiration and expiration were minimized (Figure 4). We therefore believe the observed mask surface area change and rate of change, as well as elastic/rheological properties and possibly minimal residual leakage should account for the difference between inspiration and expiration, as well as for the resistance—flow looping. Altogether, the observed flow dependence of the mask resistance was probably of trivial relevance under conditions of quiet breathing, and the usefulness of K2 may be questioned in such circumstances. On the other hand, it was thought that it may be of help in more fully describing the mask mechanical properties and contribution to increasing work of breathing during exercise, where ventilation is significantly increased. Furthermore, it may be worth applying to a more detailed analysis of V′ – R relationship during cough which is known to develop several folds increase in expiratory flow and thus promotes long distance aerosol dispersion through turbulent airflow (Sommerstein et al., 2020).

It is a common observation that, once settled on his face, a subject may become oblivious to the presence of the mask; awareness of it resumes when more ventilation is required, for instance, by walking up the stairs. In a recent review of the literature assessing face mask or respirator during exercise however (Hopkins et al., 2021), little effect of either type of equipment was reported on work of breathing or on arterial oxygen saturation in healthy individuals. Dyspnoea did not appear to be increased when external resistances were added to the breathing equipment in subjects exercising in laboratory-controlled conditions. On the other hand, wearing a face mask may increase both respiratory load and dead space. Rebreathing may lead to minute increase in alveolar PCO_2_, a strong determinant of the sensation of air hunger (Banzett et al., 1990). Generally, various physiological mechanisms may be triggered by wearing a face mask, altering breathing and breathing sensation. For instance, the increase in face skin temperature was found to be associated with breathing discomfort in healthy subjects performing treadmill exercise, while wearing FFP respirators (Kim et al., 2016). The possibility may not be excluded that face skin temperature may also increase as a result of a surgical mask and impact on breathing or breathing related sensations. That a neural pathway exists from facial thermoreceptors to respiratory motoneurons is demonstrated by the long known trigeminal diving reflex (Widdicombe, 2006). It is interesting that refinement in mask design may include assessment devices for breathing conditions (Liu et al., 2019).

This study indicates that surgical mask airflow resistance may be reproducibly measured under conditions of tidal breathing, when the mask has been carefully sealed on the dummy head. We are aware that the mask mechanics remains to be evaluated under real-life conditions; i.e., while simply attached to the back of the head and ears. In this regard, the current estimates of surgical mask maximal mechanical load may be quite helpful to test different ways of attaching the face protections. A further limitation of the current model relates to the range of flow limited to tidal breathing. This however may be improved to generate ventilation regimens that encompass those occurring at exercise or during such respiratory manoeuvres as coughing or sighing, i.e., large air flow conditions favoring aerosol particle dispersion (Sommerstein et al., 2020). We also surmise that the current model could be implemented to study the change in resistance when the mask is exposed to hot and humidified air flow over a prolonged period, so as to more precisely estimate the mechanical deterioration with time. Studies may also be developed to compare different types of face protection. The more recent knowledge that aerosol dispersion of viral particles may be a significant contributor to COVID-19 transmission further deserves detailed assessments of less filterable and more resistive material such face piece respirators.

Altogether, the face mask tolerance *in vivo* is likely to depend not only on its own mechanical properties but also on other effects, such as the added dead space, let alone the respiratory condition of the subject. Improving breathability of face mask is critical to insure compliance with the protection and therefore to prevent dissemination of air borne viral infections.

## Data Availability Statement

The original contributions presented in the study are included in the article/supplementary material, further inquiries can be directed to the corresponding author.

## Author Contributions

BD, FM, CD, and SD-A have prepared the project of this study. BD constructed physical analog and managed with CD preparatory phase of the study, assured technical assistance during resistance measures, and performed data collection. SD-A performed statistical analysis. FM, SD-A, and BD prepared the draft of manuscript. All authors contributed to the article and approved the submitted version.

## Funding

This work was supported by Ministry of Higher Education and Research of France (Ministère de l’Enseignement supérieur et de la Recherche) under contract EA 3450 DevAH.

## Conflict of Interest

The authors declare that the research was conducted in the absence of any commercial or financial relationships that could be construed as a potential conflict of interest.

## Publisher’s Note

All claims expressed in this article are solely those of the authors and do not necessarily represent those of their affiliated organizations, or those of the publisher, the editors and the reviewers. Any product that may be evaluated in this article, or claim that may be made by its manufacturer, is not guaranteed or endorsed by the publisher.
